# Observational evidence and strength of evidence domains: case examples

**DOI:** 10.1186/2046-4053-3-35

**Published:** 2014-04-23

**Authors:** Maya O’Neil, Nancy Berkman, Lisa Hartling, Stephanie Chang, Johanna Anderson, Makalapua Motu’apuaka, Jeanne-Marie Guise, Marian S McDonagh

**Affiliations:** 1 Scientific Resource Center for the Agency for Healthcare Research and Quality Effective Health Care Program, Portland VA Research Foundation, Portland VA Medical Center, Portland, OR 97239, USA; 2 Research Triangle Institute and University of North Carolina Evidence-based Practice Center, Research Triangle Park, NC 27709-2194, USA; 3 Department of Pediatrics and Evidence-based Practice Center, University of Alberta, Edmonton, AB T6G 1Z1, Canada; 4 Center for Outcomes and Evidence, Agency for Healthcare Research and Quality, Rockville, MD 20850, USA; 5 Department of Medical Informatics and Clinical Epidemiology and Evidence-based Practice Center, School of Medicine, Oregon Health & Science University, Portland, OR 97239-3098, USA

**Keywords:** Systematic reviews, Observational studies, Non-randomized studies, Strength of evidence, AHRQ Effective Health Care Program, Integrative reviews, Mixed methods reviews, Cross-sectional studies, Case series, Case-control studies

## Abstract

**Background:**

Systematic reviews of healthcare interventions most often focus on randomized controlled trials (RCTs). However, certain circumstances warrant consideration of observational evidence, and such studies are increasingly being included as evidence in systematic reviews.

**Methods:**

To illustrate the use of observational evidence, we present case examples of systematic reviews in which observational evidence was considered as well as case examples of individual observational studies, and how they demonstrate various strength of evidence domains in accordance with current Agency for Healthcare Research and Quality (AHRQ) Evidence-based Practice Center (EPC) methods guidance.

**Results:**

In the presented examples, observational evidence is used when RCTs are infeasible or raise ethical concerns, lack generalizability, or provide insufficient data. Individual study case examples highlight how observational evidence may fulfill required strength of evidence domains, such as study limitations (reduced risk of selection, detection, performance, and attrition); directness; consistency; precision; and reporting bias (publication, selective outcome reporting, and selective analysis reporting), as well as additional domains of dose-response association, plausible confounding that would decrease the observed effect, and strength of association (magnitude of effect).

**Conclusions:**

The cases highlighted in this paper demonstrate how observational studies may provide moderate to (rarely) high strength evidence in systematic reviews.

## Background

Historically, systematic reviews of healthcare interventions have focused on randomized controlled trials (RCTs), primarily because randomization is intended to control for both known and unknown confounders, resulting in the ability to attribute differences between groups to the intervention under study. Increasingly, systematic reviews of healthcare interventions include observational studies when RCT evidence is considered inadequate; trials may be considered infeasible or unethical, do not report long-term or less common serious outcomes (particularly harms), or do not reflect use in real-world settings in terms of populations included, comparisons made, or how the intervention is applied. We define observational studies according to the definition used in the Agency for Healthcare Research and Quality’s (AHRQ’s) Evidence-based Practice Center (EPC) guidance on using observational studies in systematic reviews: 'Observational studies of interventions are defined herein as those where the investigators did not assign exposure; in other words, these are nonexperimental studies. Observational studies include cohort studies with or without a comparison group, cross-sectional studies, case series, case reports … and case-control studies’ [1].

To support and improve use of observational evidence, we present case examples of systematic reviews in which observational evidence was considered as well as case examples of individual observational studies demonstrating various strength of evidence domains. This paper illustrates how the current AHRQ methods guidance can be applied to observational evidence.

## Methods

Several chapters of the AHRQ EPC *Methods Guide* provide guidance on the role of observational studies [2-5]: when to include evidence from observational studies, how to assess harms, how to assess the risk of bias of individual studies, and how to assess the strength of an entire body of evidence. Systematic reviews that included observational studies and individual observational studies were solicited via informal discussions with AHRQ EPC members comprising the AHRQ EPC Methods Workgroups [6] in 2012 to 2013. We analyzed the content of these reviews and studies in order to provide examples of how observational studies may be used to support decision-making, particularly in the absence of high quality or applicable trial data, based on the AHRQ methods guidance [2,7].

## Results and discussion

### When to include observational studies in systematic reviews of healthcare interventions

A systematic review provides evidence to inform decision-making. While some may argue that decisions should only be made on high strength evidence, many acknowledge the necessity of decision-making even in the face of imperfect evidence. With this understanding, the AHRQ EPC guidance recommends that systematic reviews provide the best available evidence to help decision-makers [7]. Due to confounding, observational evidence generally provides lower strength evidence than RCTs. However, in some cases, this may be the best available evidence.

Norris *et al*. [1] proposed that reviewers include observational studies in a systematic review when conclusions from RCT bodies of evidence are inconsistent, indirect, imprecise, inapplicable, or not generalizable. Similarly, the Grading of Recommendations Assessment, Development and Evaluation (GRADE) Working Group guidance states that the inclusion of observational studies may be warranted, as a complement to RCTs, to provide data sequential to the information provided by RCTs (for example, in the case of longer-term data on outcomes), or as a replacement for RCT evidence when no RCT evidence exists [8]. They highlight the frequent need for inclusion of observational studies for questions related to directness (that is, when the populations examined in RCTs are too different from the population of interest to generalize the findings). The Cochrane Collaboration provides similar recommendations [9]. While all three groups support circumstantial use of observational studies in a systematic review, all also note concern about the higher risk of bias associated with observational studies compared to RCTs.

While Higgins *et al*. [10] provided recommendations for *a priori* inclusion criteria, they highlighted the complexities in making such decisions before other information is known (for example, search yield or risk of bias of included RCTs). They described a lack of consensus among authors of systematic reviews as to whether absolute pre-specified criteria should be followed or if a sequential approach to determining and modifying 'best evidence’ throughout the course of the review is preferable in some instances. A decision framework for identifying best evidence was described by Treadwell *et al*. [7], including how to prioritize available evidence for inclusion and addressing the potential need for including observational study evidence in reviews.

Chou *et al*. [3] provided recommendations for including observational studies when assessing harms, particularly under the conditions described above (when trials are lacking, generalizability is uncertain). The authors also noted that risk of bias from confounding may be lower when investigating unexpected harms and in cases of rare or long-term harms where observational studies may actually provide the best evidence. Overall, the available guidance on when to include observational studies in systematic reviews of healthcare interventions describes decisions influenced by specific questions of interest and clinical contexts in order to improve the validity and relevance of systematic reviews to decision-making.

### Case examples: observational studies as 'best evidence’ in systematic reviews

In some reviews of healthcare interventions, RCTs were considered infeasible or unethical, lacked generalizability, or were poor quality or insufficient in number. In these examples, observational evidence may provide only low strength of evidence, but provide the best available evidence to help decision-makers [7].

#### Feasibility or ethical concerns

A systematic review examining evidence on cesarean delivery on maternal request (CDMR) [11] sought to compare planned cesarean delivery in the absence of medical or obstetric indications with planned vaginal delivery. However, research involving pregnant women raises a unique set of feasibility and ethical concerns and the preferences of the pregnant woman must be considered. An RCT would have provided the most rigorous evaluation of the benefits specific to route of delivery, but because data on women randomized to a particular birth plan were not available, the reviewers sought evidence from observational studies that reported the actual (rather than planned) route of delivery.

#### Lack of generalizability of randomized controlled trials (RCTs)

Another review focused on the effectiveness of atypical antipsychotic drugs for schizophrenia, bipolar affective disorder, and other mental health disorders [12]. The review included observational studies for the assessment of effectiveness outcomes (for example, employment) and harms. In spite of a fairly large number of head-to-head comparison RCTs for efficacy and effectiveness, public comments received from advocacy groups and the pharmaceutical industry indicated significant concerns about the generalizability of the trials. In investigating these concerns, the review team found that the dosing in some trials was outside the effective range and therefore potentially less likely to result in adverse events than in real-life clinical practice (usually conducted before or soon after the US Food and Drug Administration approval of the newest drug in the trial). The review team also found that many trials narrowly defined patient populations, including only patients with little comorbidity and those who used few or no concomitant medications. Minorities, older patients, and the most seriously ill patients were underrepresented. The participants were generally young (20s and 30s) with mostly moderate symptoms. As a result, the review authors made a decision to include comparative observational studies that reported benefit outcomes in a subsequent update of the report as these studies were better able to address questions of effectiveness, generalizability, and harms [13].

#### Limited RCT data

Two AHRQ reviews [14,15] on behavioral interventions for autism spectrum disorders (in children, adolescents, and young adults) included observational studies as well as trials, due to the small number of available trials. Further, the trials reported on limited intervention types and outcomes, and in one of the reviews were of low quality. The review teams included reports of at least ten children to obtain evidence on response to treatment in very short timeframes and under very tightly controlled circumstances. These studies did not provide information on longer-term or functional outcomes, nor were they ideal for determining external validity without multiple replications. In both reviews, the inclusion of observational data did not significantly improve the strength of evidence for treatment effectiveness; however, the authors chose to include them to highlight the need for stronger studies to increase the strength of evidence. While the inclusion of observational evidence may increase the strength of evidence for certain outcomes, in other cases it may be included as a way to assure that all relevant data have been considered in a 'best evidence’ approach to decision-making, or to highlight future research needs, as in this example. A systematic review of interventions for cryptorchidism [16], described in greater detail later in this paper, provides an example of observational studies increasing the strength of evidence in a systematic review when RCT data are not available.

### Study limitations of observational studies

Lack of randomization can bias observational studies. Specifically, potential confounding and selection bias mean treatment and control group differences cannot be assumed to result from the intervention. The *Cochrane Handbook* defines selection bias as 'systematic differences between baseline characteristics of the groups that arise from self-selection of treatments, physician-directed selection of treatments, or association of treatment assignments with demographic, clinical, or social characteristics. It includes Berkson’s bias, nonresponse bias, incidence-prevalence bias, volunteer/self-selection bias, healthy worker bias, and confounding by indication/contraindication (when patient prognostic characteristics, such as disease severity or comorbidity, influence both treatment source and outcomes)’ [17]. Additional sources of bias in observational studies can arise because of the data source, study design, and analytic method. Certain characteristics of observational studies, such as using a population-based new-user design or using statistical adjustment or matching procedures, may decrease the risk of bias, which can increase confidence in the results. It is generally considered impossible to completely mitigate the potential for bias associated with observational studies through study design or analytic method because residual unidentified confounding factors can rarely be ruled out, and statistical adjustment or matching procedures are often inadequate. Other newer statistical techniques are complicated and imperfect, although can help mitigate some study design flaws common to observational studies (for example, new-user design [18] and high-dimensional propensity score adjustment [19,20]).

Potential sources of bias in observational studies are well documented [9,21]. The AHRQ EPC *Methods Guide* provides guidance for assessing risk of these biases in observational studies [4]. As this paper and others [5,10,22] note, there is not an agreed-upon standard for assessing risk of bias for observational studies, although examples of commonly used assessment tools include the Newcastle-Ottawa Scale, Downs and Black tool [23] (see Deeks *et al*. [24] for a summary and review), and the RTI item bank [25].

### Strength of evidence domains and observational evidence

In addition to the inherent biases from lack of randomization, observational studies are subject to the same risks of other biases as RCTs. Thus, observational studies are considered to have greater study limitations than RCTs. Because the study limitations in the body of evidence is considered the starting point for assessing confidence in the findings of a body of evidence (along with directness, precision, and consistency), the AHRQ EPC *Methods Guide* recommends that findings from a body of observational studies generally start as low strength due to the 'higher risk of bias attributable to a lack of randomization (and inability of investigators to control for critical confounding factors)’ [2], but may be increased under certain conditions. Specifically, the AHRQ EPC *Methods Guide* states that 'EPCs may move up the initial grade for strength of evidence based on observational studies to moderate when the body of evidence is scored as low or medium study limitations, based on controls for risk of bias through study conduct or analysis. Similarly, EPCs may initially grade the strength of evidence as moderate for certain outcomes such as harms or certain key questions, when observational study evidence is at less of a risk for study limitations because of a lower risk of bias related to potential confounding. Also, EPCs may well decide that, after assessing the additional domains, the overall strength of evidence of a body of observational studies can be upgraded to moderate (although rarely high)’ [2], page 20.

The required domains for assessing strength of evidence according to the AHRQ EPC *Methods Guide* are study limitations (reduced risk of selection, detection, performance, attrition, and reporting bias); directness; consistency; precision; and reporting bias (publication, selective outcome reporting, and selective analysis reporting). The AHRQ EPC *Methods Guide* specifically defines three additional domains applicable to observational studies that, if met, would potentially warrant increasing the strength of evidence rating. These three additional domains include dose-response association, plausible confounding that would decrease the observed effect, and strength of association (magnitude of effect). The following studies are provided to demonstrate what these strength of evidence factors look like in real-world examples.

### Case examples: strength of evidence domains for observational studies

In some cases the observational evidence demonstrates criteria that elevate the strength of evidence. However, because the examples are real-world case examples, not theoretical examples designed to neatly demonstrate all domains, not all included examples would result in increased ratings of strength of evidence. Rather, because we hope to advance training for others conducting systematic reviews, we illustrate how the examples demonstrate specific strength of evidence domains.

A Cochrane review [26] investigated the effectiveness of bicycle helmets in reducing head, brain, and facial injuries (Table 1). No RCTs or cohort studies were found; therefore, only case-control studies were included in the review. The reviewers limited studies to those that included active case ascertainment; a determination of exposure and helmet use at the time of bicycle crash; proper control group selection; and elimination or control of factors such as selection bias, observation bias, and confounding. Five studies included in the review showed a significantly decreased likelihood of head and brain injury during a bicycle crash with helmet use. Summary odds ratios (ORs) and 95% confidence intervals (CIs) were calculated for these studies. Helmet use was associated with a reduced likelihood of head injury by 69% (OR 0.31, 95% CI 0.26 to 0.37) and brain injury by 69% (OR 0.31, 95% CI 0.23 to 0.42). A protective association of 64% (OR 0.36, 95% CI 0.26 to 0.49) was found for upper facial injury and a protective association of 65% (OR 0.35, 95% CI 0.24 to 0.50) was found for middle facial injury. Additionally, one study using a population-based control group found a protective association of 85% (OR 0.15, 95% CI 0.07 to 0.29) and 88% (OR 0.12, 95% CI 0.04 to 0.40) for head and brain injury, respectively.

**Table 1:** Systematic review case example: helmets for preventing head, brain, and facial injuries in bicyclists

**Strength of evidence factors**	**Strength of evidence domains**
Required domains	Study limitations:
	• Reduced risk of selection bias: controls from the same population as cases
	• Reduced risk of detection bias: independent outcome assessors
	Consistency: consistent direction of effect for the primary outcome observed across multiple studies
	Precision: precise effect estimate across included studies
Additional domains	Strength of association: large magnitude of effect

The evidence that helmets reduce brain, head, and facial injuries presented from case-control studies in this review is strengthened by various factors despite the nonexperimental study designs. First, the included studies were classified as having low risk of bias based on criteria specific to case-control studies, because controls were selected from the same population as cases, injuries were verified by medical records, and ascertainment of exposure was equivalent for case and control groups. Additionally, there was a consistent direction of effect for the primary outcome of head injury in all five studies. Finally, a large magnitude of effect and precise estimate was seen across all included studies: the protective effects of helmet use on head, brain, and facial injury ranged from 64% to 88%.

An AHRQ systematic review on evaluating and treating cryptorchidism [16] assessed the effectiveness of imaging for identifying and correctly locating testicles; the use of hormonal stimulation for treatment planning and hormones for achieving testicular descent; and choices among surgical treatments, including surgical approach (open versus laparoscopic) (Table 2). The goal of an intervention for cryptorchidism is to move the undescended testicle to a normal position in the scrotum in the safest and least invasive way possible. Participants included prepubescent males with cryptorchidism. Studies included all designs except case reports. Treatment options examined required an appropriate comparison arm and an initial trial of hormone therapy to elicit testicular descent or surgical repair.

**Table 2:** Systematic review case example: evaluation and treatment of cryptorchidism

**Strength of evidence factors**	**Strength of evidence domains**
Required domains	Study limitations:
	• Reduced risk of performance bias: objective primary outcome
Additional domains	Strength of association: large magnitude of effect

Of 26 included surgical treatment studies, five were RCTs, one was a prospective cohort, and the rest were retrospective cohort studies rated as having high risk of bias. Decisions about method of surgical repair were made based on clinical presentation (for example, location of the affected testicle) and patient/parent preferences, and not with the intent of comparing the effectiveness of the procedures in comparable groups of patients, making the comparison groups essentially different. Because these studies did not control for initial testicular location, the results can only be interpreted as providing noncomparative data on outcomes in groups with differing clinical presentations treated surgically. The systematic review authors elected to use was based on a historical control group given the known natural history of the condition. Given the low rate of spontaneous testicular descent, the strength of the evidence was considered high because of the large magnitude of effect for an objective outcome when compared with a historical control group. The weighted success rate for all three surgical approaches exceeded 75%, with an overall reported rate of 79% for one-stage Fowler-Stephens (FS) orchiopexy procedure, 86% for two-stage FS orchiopexy procedure, and 96.4% for primary orchiopexy. Due to variation in surgical repair techniques (for example, open versus laparoscopic approaches), which are often guided by testicular location, patient/parent preferences, surgeon skill, and recovery time, included studies were not able to provide comparative evidence for the relative effectiveness of these techniques. Although only retrospective cohort studies examined primary orchiopexy for the outcome of testicular decent, the overall effectiveness of this type of surgical treatment was rated as high strength of evidence due to the magnitude of effect when compared with historical controls.

As with many surgical interventions, for treatment of obesity, it is neither feasible nor ethical to randomize bariatric surgery in comparison to conventional nonsurgical obesity interventions. Sjöström *et al*. [27] published an observational study of the effects of bariatric surgery on mortality and is an example of a methodologically strong study (Table 3). The study was prospective and adequately powered by including a large sample across multiple clinical settings (n = 4,047 participants from 480 clinics and 25 surgical departments). The study was designed so that surgical participants were prospectively matched to controls on 18 potentially important confounding variables. Minimal exclusion criteria allowed for a population reflecting the general population of obese patients and included those with such comorbidity as histories of hypertension, diabetes, stroke, and myocardial infarction. The outcome of interest was all-cause mortality and therefore less risk of performance bias. Although participant and provider awareness of treatment condition could influence behavior, there is less concern of performance bias because of the objective nature of the outcome. Additionally, although cause of death was determined by outcome assessors and could be less objective than simply recording mortality from death records, two blinded independent outcome assessors reviewed all autopsies and a third assessor reviewed the autopsy prior to final determination of cause of death. Other outcomes of interest such as weight loss were also reported, and the direction of effect was consistent across outcomes. In addition to objective outcomes assessed by blinded outcome assessors, a 15-year follow-up made mortality data available for virtually all (99.9%) participants. Despite lack of randomization or additional corroborating studies, the strong methods employed in this study would warrant a higher strength of evidence rating (for example, moderate strength of evidence) because of the low risk of bias (including low risk of selection, detection, attrition, and reporting bias) as well as direct and precise results.

**Table 3:** Primary study case example: effects of bariatric surgery on mortality in Swedish obese subjects

**Strength of evidence factors**	**Strength of evidence domains**
Required domains	Study limitations:
	• Reduced risk of selection bias: matched sample to address potentially influential confounding variables, minimal exclusion criteria, prospective study design, very large sample size
	• Reduced risk of detection bias: objective outcome and independent outcome assessors
	• Reduced risk of attrition bias: high rate of follow-up
	• Reduced risk of reporting bias: *a priori* protocol identifying primary outcomes
	Directness: minimal exclusion criteria from a large sample at many hospitals and clinics provided direct evidence of key outcomes for the population of interest
	Precision: adequately powered study resulted in a precise effect estimate

Harms associated with cancer treatments can be difficult to evaluate based on randomized trial results, and evidence of harms is often based on observational study designs. The two studies described here used case-control study designs. Neglia and colleagues [28] investigated primary neoplasms of the central nervous system as a harm associated with radiation therapy treatment for childhood cancer using cases and controls from a cohort of about 14,000 5-year childhood cancer survivors who had received radiation as part of their prior cancer treatment. In this study, 116 cases of primary neoplasms were identified. Each case was matched to four control subjects by age, sex, and time since original cancer diagnosis. A second study [29] examined the risk of ischemic heart disease as a harm associated with radiation therapy for breast cancer. This study included 963 cases with major coronary events and 1,205 controls selected at random from all eligible women in the study population. Eligibility criteria included receiving a cancer diagnosis between the years of 1958 and 2001, being less than 70 years of age, and having received radiotherapy.

These studies both reported a dose-response relationship between the outcome and the mean dose of radiation therapy (Table 4). In childhood cancer survivors, a linear dose-response relationship was observed between primary neoplasms of the central nervous system (glioma and meningioma) and radiation dose (gray; Gy). An increased risk for development of subsequent glioma (adjusted OR 6.78, 95% CI 1.54 to 29.7) and meningioma (adjusted OR 9.94, 95% CI 2.17 to 45.6) and for all tumors combined (OR 7.07, 95% CI 2.76 to 18.1) was found with level of exposure to radiation therapy. A dose-response relationship was also observed for glioma (slope = 0.33, 95% CI 0.07 to 1.71), for meningioma (slope = 1.06, 95% CI 0.21 to 8.15), and for all tumors combined (slope = 0.69, 95% CI 0.25 to 2.23). Among women who received radiation therapy for breast cancer, major coronary events (that is, myocardial infarction, coronary revascularization, or death from ischemic heart disease) increased linearly with increasing radiation dose. The rate of major coronary events increased linearly by 7.4% (95% CI 2.9 to 14.5) per mean radiation dose (Gy).

**Table 4:** Primary study case examples: new primary neoplasms of the central nervous system in survivors of childhood cancer/risk of ischemic heart disease in women after radiotherapy for breast cancer

**Strength of evidence factors**	**Strength of evidence domains**
Additional domains	Dose-response association: there was a linear association between harm and amount of radiation exposure

Although both of these studies were observational designs, the dose-response relationships observed between the intervention and the harm could be considered when rating strength of evidence. When the effect of an intervention increases proportionally to the dose of the intervention, we can be more confident that the observed effect is in response to the intervention and not the result of bias or confounding. As noted in the AHRQ EPC *Methods Guide*, evidence from single studies cannot meet criteria for consistency, and particularly when paired with a small sample size, may warrant an 'insufficient’ strength of evidence rating. Similarly, evidence meeting only some of the strength of evidence criteria should not be upgraded [2]. However, because these studies are being used to assess potential harms, the strength of evidence may initially be graded as moderate, as per AHRQ EPC methods guidance.

## Conclusions

In this paper, we provided cases that highlight: 1) systematic reviews of observational evidence included to fill gaps in RCT evidence; and 2) systematic reviews of observational studies as well as primary observational studies that demonstrate strength of evidence domains as described in the AHRQ EPC *Methods Guide*. These cases are meant to inform the decision to include/exclude observational studies and how to evaluate their strength of evidence in systematic reviews.

In general, we can be more confident in the results of observational studies when design or analyses have minimized the potential for common sources of bias, results are precise and consistent, and when we observe a large strength of association, a dose-response association, or plausible confounding very likely to decrease the observed effect. Importantly, of all the examples of strong observational studies solicited for this project, we did not identify any additional strength of evidence factors not already included in the AHRQ EPC *Methods Guide*, providing support for the comprehensiveness of this and other similar guidance. These strength of evidence domains are often specific to clinical topics and individual study factors warrant careful consideration before upgrading an observational study body of evidence, as noted in the current AHRQ EPC *Methods Guide* on strength of evidence [2]; however, our case examples show instances where studies should not be automatically excluded because they are not RCTs. Further identification and description of cases where observational studies have contributed to higher strength of evidence ratings in a systematic review of healthcare interventions would be beneficial. Future research could expand upon these case examples to include demonstrations of how to conduct risk of bias assessment and strength of evidence ratings for observational studies.

## Abbreviations

AHRQ: Agency for Healthcare Research and Quality; CDMR: Cesarean delivery on maternal request; CI: Confidence interval; EPC: Evidence-based Practice Center; FS: Fowler-Stephens; GRADE: Grading of Recommendations Assessment Development and Evaluation; Gy: Gray; OR: Odds ratio; RCT: Randomized controlled trial.

## Competing interests

The authors declare that they have no competing interests.

## Authors’ contributions

MO participated in conception and design of the project, and drafted the manuscript. NB, LH, SC, and JMG helped analyze and interpret project material, and critically revised the manuscript. JA and MM helped analyze project material and helped to draft the manuscript. MSM participated in conception and design of the project, and helped to draft the manuscript. All authors read and approved the final manuscript.
